# Supplementary material for: DNA methyltransferase inhibition upregulates MHC-I to potentiate cytotoxic T lymphocyte responses in breast cancer
Source: Nat Commun. 2018 Jan 16;9:248. doi: 10.1038/s41467-017-02630-w (PMC5770411; doi:10.1038/s41467-017-02630-w)
Supplement: Supplementary file 1 — Supplementary Information [file 41467_2017_2630_MOESM1_ESM.pdf]

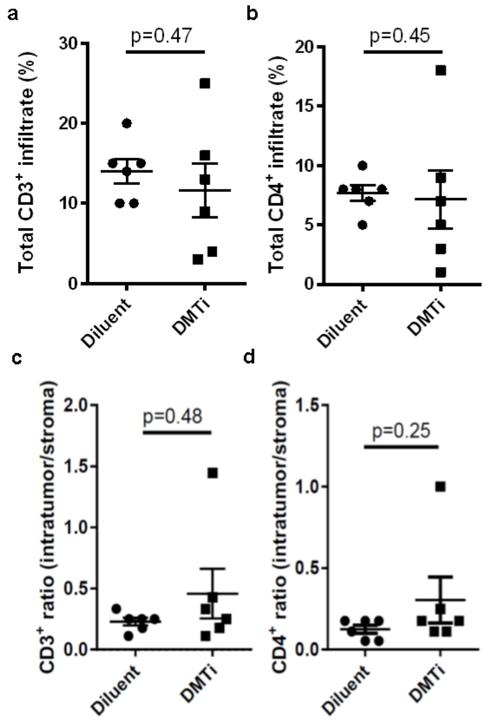

Supplementary Figure 1: DMTi treatment exerts minimal effects on CD3+ or CD4+ infiltration in MMTV-Neu tumors. Evaluation of CD3+ (**a & c**) or CD4+ (**b & d**) infiltration in MMTV-Neu tumors by IHC under in vivo administration of Diluent vs DMTi. The result was illustrated as percentage of CD3+ (**a**) or CD4+ (**b**) over the whole tumor section, or the ratio of CD3+ (**c**) or CD4+ (**d**) infiltration between intra-tumor and stroma compartments. P-value is calculated using unpaired t test. All data are means + SEM. Each data point represents one mouse.

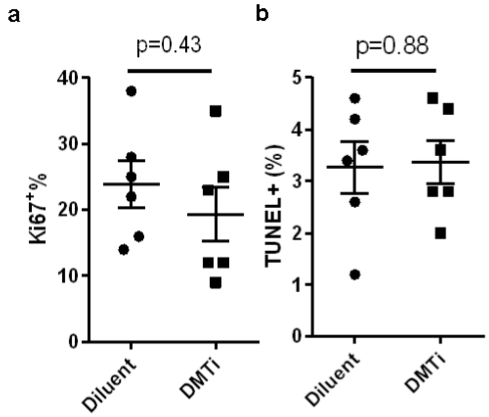

Supplementary Figure 2: Molecular changes in proliferation and apoptosis in tumors following guadecitabine treatment. **a.** Ki67 staining and **b.** TUNEL staining in MMTV-Neu tumors 7 days after a single course of guadecitabine therapy. Although not significant, a minor decrease in proliferation was observed following treatment, as assessed by Ki67.

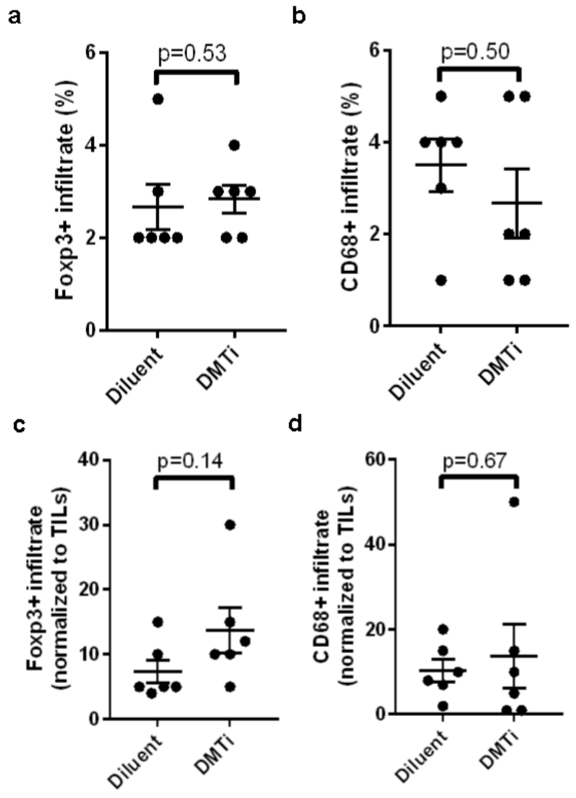

Supplementary Figure 3: No change in Foxp3+ or CD68+ infiltrate in tumors following guadecitabine treatment. **a.** Immunohistochemical quantitation of Foxp3+ infiltrate (% of total nuclei in field of view) and **b.** CD68+ infiltrate staining in MMTV-Neu tumors 7 days after a single course of guadecitabine therapy. Markers were also assessed as a percent of total TILs in **c.** and **d.**, respectively. Although not significant, a minor increase in Foxp3+ cells, when assessed as percent of total TILs, was observed following treatment.

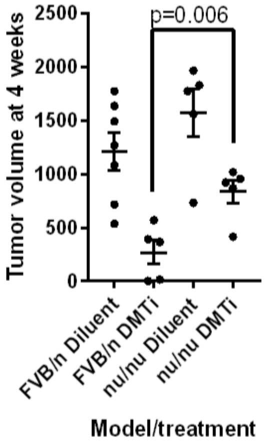

Supplementary Figure 4: Comparison of guadecitabine efficacy in T cell-deficient and immunocompetent hosts. MMTV-neu tumors were orthotopically implanted into syngeneic (FVB/n) or athymic nu/nu mice and allowed to reach 100-300mm^3 prior to treatment with diluent control or guadecitabine daily by IP injection for 3 days. Tumor volumes were measured until 4 weeks. Data are expressed as final tumor volume at 4 weeks (or at humane endpoint, if occurring prior to 4 weeks). Although guadecitabine was effective in both models, there was a greater beneficial effect in immunocompetent hosts, suggesting a role for T cell-mediated anti-tumor immunity.

Supplementary Table 1: Methylated and unmethylated primer sequences for mouse H2-D1

Primers	DNA sequence (5’->3’)
**Unmethylated 1 forward**	GAGGAGTTTTGGTATATTTTTGTTG
**Unmethylated 1 reverse**	CCCTTAACTTTCTATATTTCCCACT
**Unmethylated 2 forward**	GAGGAGTTTTGGTATATTTTTGTTG
**Unmethylated 2 reverse**	CCCTTAACTTTCTATATTTCCCACT
**Unmethylated 3 forward**	GAGGAGTTTTGGTATATTTTTGTTG
**Unmethylated 4 reverse**	CCCTTAACTTTCTATATTTCCCACT
**Methylated 1 forward**	GAGGAGTTTCGGTATATTTTTGTC
**Methylated 1 reverse**	TAACCCTTAACTTTCTATATTTCCCG
**Methylated 2 forward**	AGGAGTTTCGGTATATTTTTGTCG
**Methylated 2 reverse**	CCTTAACTTTCTATATTTCCCGCT
**Methylated 3 forward**	GAGGAGTTTCGGTATATTTTTGTC
**Methylated 3 reverse**	TAACCCTTAACTTTCTATATTTCCCG

Supplementary Table 2: Primer sequences for qRT-PCR

Primers	DNA sequence (5’->3’)
**H2-D1-1 forward**	AGGAACCTGCTCGGCTACTA
**H2-D1-1 reverse**	GCCCTGAACGAAGACCTGAA
**H2-D1-2 forward**	TACCTGAAGAACGGGAACGC
**H2-D1-2 reverse**	CCCTGACCTGGCAGTTGAAT
**H2-D1-3 forward**	AGGTGAAGTCACCCTGAGGT
**H2-D1-3 reverse**	ATCTGTGGTGGTGCCTCTTG
**Gapdh forward**	AGGTCGGTGTGAACGGATTTG
**Gapdh reverse**	TGTAGACCATGTAGTTGAGGTCA
**Cxcl9 forward**	GGCTCGCAGGGATGATTTCAA
**Cxcl9 reverse**	CCAAGTGCTGCCGTCATTTTC
**Cxcl10 forward**	GGAGTTCGAGGAACCCTAGTG
**Cxcl10 reverse**	GGGATTTGTAGTGGATCGTGC
**Cxcl11 forward**	GGCTTCCTTATGTTCAAACAGGG
**Cxcl11 reverse**	GCCGTTACTCGGGTAAATTACA
