# Supplementary material for: DNA methyltransferase inhibition upregulates MHC-I to potentiate cytotoxic T lymphocyte responses in breast cancer
Source: Nat Commun. 2018 Jan 16;9:248. doi: 10.1038/s41467-017-02630-w (PMC5770411; doi:10.1038/s41467-017-02630-w)
Supplement: Supplementary file 3 — Description of Additional Supplementary Files [file 41467_2017_2630_MOESM3_ESM.pdf]

Description of Additional Supplementary Files

File Name: Supplementary Data 1
Description: NanoString Pan-Cancer Immune panel normalized transcript count data from MMTV-neu tumors treated with guadecitabine or vehicle control (linear scale).
